# Equilibrium States in Open Quantum Systems

**DOI:** 10.3390/e20060441

**Published:** 2018-06-06

**Authors:** Ingrid Rotter

**Affiliations:** Max Planck Institute for the Physics of Complex Systems, D-01187 Dresden, Germany; rotter@pks.mpg.de

**Keywords:** open quantum systems, non-Hermitian Hamilton operator, equilibrium state

## Abstract

The aim of this paper is to study the question of whether or not equilibrium states exist in open quantum systems that are embedded in at least two environments and are described by a non-Hermitian Hamilton operator H. The eigenfunctions of H contain the influence of exceptional points (EPs) and external mixing (EM) of the states via the environment. As a result, equilibrium states exist (far from EPs). They are different from those of the corresponding closed system. Their wavefunctions are orthogonal even though the Hamiltonian is non-Hermitian.

## 1. Introduction

In many recent studies, quantum systems are described by a non-Hermitian Hamilton operator H. Mainly, only the eigenvalues of H are considered because the main interest of these studies are in receiving an answer to the question of whether the eigenvalues of the non-Hermitian Hamiltonian are real or complex [1]. Additionally, the conditions for changing from real to complex eigenvalues and vice versa are considered in many papers. In other studies, calculations for realistic small quantum systems are performed by using the complex scaling method [2]. Recently, topological insulators are also considered in the presence of non-Hermiticity [3,4,5]. In the description of realistic systems, the non-Hermiticity of the Hamiltonian arises from embedding the system into an environment as explained in References [6,7]. It is derived from the full Hamiltonian, describing a system together with the environment which is, of course, Hermitian.

In order to describe the properties of a realistic quantum system, one needs not only the eigenvalues of the Hamiltonian but also its eigenfunctions. This is nothing but the longtime experience obtained from standard quantum mechanics calculations performed with Hermitian operators. There is no reason why this should be different in the description of a system by means of a non-Hermitian Hamiltonian.

The characteristics of the eigenvalues together with those of the eigenfunctions of a non-Hermitian Hamiltonian, H, are, so far, only studied for a small open quantum system in the papers of only one group, see e.g., Reference [6]. Their theoretical results are compared with those known experimentally [7]. These theoretical studies are performed in the same manner as those in standard quantum mechanics, with the only difference being that the Hermitian Hamiltonian *H* is replaced by a non-Hermitian operator H. The results, therefore, allow us to find a direct answer to the question of whether or not it is necessary to describe a system (in a special situation) by a non-Hermitian Hamilton operator. Additionally, the results show that a unique answer does not exist; it will depend on the conditions under which the system is considered.

Of special interest is the challenging question of whether or not an equilibrium state exists in an open quantum system that is embedded in at least two environments. The challenge of this question arises from the fact that it combines two conflicting concepts. On the one hand, the system is open meaning that its properties are not fixed and will vary under the influence of the environment. On the other hand, the system is expected to be in equilibrium. It is therefore no wonder that this question is not at all considered, up to now.

The present paper is aimed at a study of this question in the framework of the non-Hermitian formalism. For this purpose, we will sketch some results obtained by using the formalism worked out in Reference [6]. We will then consider the parametric evolution of an open quantum system and the possibility to form an equilibrium state. In any case, such a state is expected to be different from the equilibrium state of the corresponding closed system (described by the Hermitian Hamilton operator *H*).

The paper is organized in the following manner. In Section 2, the non-Hermitian formalism is shown to differ from that of the Hermitian formalism by two basic features: (i) the existence of singular points, which are mainly called exceptional points (EPs) [8] and (ii) the possibility of a mixing of the eigenfunctions of H via the environment, usually called external mixing (EM) [9]. In Section 3, the information entropy is defined, while in Section 4, the possible formation of an equilibrium state in an open quantum system (coupled to at least two environments) is considered. Section 5 is devoted to the different aspects of the question of whether or not it is really necessary to consider the non-Hermiticity of the Hamiltonian when describing an open quantum system. Here, we consider the most common case; that the system is coupled to more than one environment. The conclusions are contained in the last Section 6. In Appendix A, a few experimental results are listed which cannot be explained in the framework of Hermitian quantum physics.

## 2. Non-Hermitian versus Hermitian Formalism

In standard quantum theory, the Hamilton operator of a many-body system is assumed to be Hermitian. Its eigenvalues are real and provide the energies of the states. The lifetimes of the states cannot be calculated directly. They are usually obtained from the probability that the particles may tunnel from inside the system to the outside. The Hermitian Hamilton operator describes a closed system, and has provided many numerical results which agree well with experimentally observed results. A few experimental results are, however, not explained, in spite of much effort. They remain puzzling (see Appendix A for a few examples).

Full information on a quantum system can be obtained only when it is observed either by a special measurement device or in a natural manner, that is by the environments in which the system is embedded. Such a system is open and is described best by a Hamilton operator which is non-Hermitian [6].

Quantum systems are usually localized in space, meaning that they have a finite extension with a characteristic shape. The environments are however infinitely extended. The environments of an open quantum system can, generally, be parametrically varied and thus allow a parameter dependent study of various properties of quantum systems. Studies of such a type are now performed theoretically and experimentally on various different open quantum systems [7]. They provided many interesting results, which are necessary for a deeper understanding of quantum physics.

From a mathematical point of view, the non-Hermitian formalism is much more complicated than the Hermitian formalism. One of the most important problems is the existence of singularities [8] that may appear in the non-Hermitian formalism. At these singular points, mainly called exceptional points (EPs), two eigenvalues of the Hamiltonian coalesce and—even more relevant for the system properties—the eigenfunctions show deviations from the expected ones in a certain finite neighborhood around these points [6,9]. The deviations are caused by nonlinearities of the involved equations at and near the EPs.

At an EP, the trajectories of the eigenvalues $E_{1,2}\equiv E_{1,2}+i\Gamma_{1,2}/2$ of the two crossing states of H [10] do not move linearly as a function of a certain parameter. The eigenvalue trajectories are rather exchanged: the trajectory of state 1 continues to move as the trajectory of state 2 and vice versa. The same holds true for the trajectories of the eigenfunctions. This trajectory of the eigenvalue is influenced by an EP not only at the position of the EP, but also in its vicinity. Here, Im($E_{i}$) may be exchanged, while the corresponding Re($E_{i}$) are not exchanged; or the other way around. The first case (exchange of the widths) can be traced to discrete states [11], which is nothing but the Landau–Zener effect, known very well (but not fully explained) in Hermitian quantum physics. The trajectories of the eigenfunctions show a behavior which corresponds to that of the eigenvalue trajectories, as illustrated first in Reference [11]. In any case, a linear motion of the eigenvalue trajectories occurs only far from an EP.

Another important problem is the fact that all of the states of the system may interact via the environment. This is a second-order process since every state is directly coupled to the environment and hence—via the environment—to another state. This process is usually called external mixing (EM) of the states [6,9].

A naturally arising question is what are the conditions under which a system can be nevertheless described by a Hermitian Hamilton operator? In other words, when it is really necessary to describe a system by means of a non-Hermitian Hamilton operator?

The main results of many studies on realistic quantum systems are the following:

(i) Far from EPs and at low level density (every state is well separated from neighboring states), the description of a system by means of a Hermitian Hamilton operator provides good results. This fact is well known from countless calculations over many years.
(ii) Near to EPs, the non-Hermitian formalism provides results which are counterintuitive. These results agree (at least qualitatively) with puzzling experimentally observed results (see Appendix A).
(iii) The EM of all states via the environment changes the eigenfunctions of the Hamiltonian even though EM is a second-order process [6,9].
(iv) In approaching an EP, the EM increases to infinity [9]. It is therefore impossible for any source (such as light) to interact with the system at an EP (for an example, see Appendix A).

The answer to the above question is therefore, that the non-Hermiticity of the Hamiltonian must be taken into account in all numerical calculations for which the influence of EPs and EM cannot be neglected. All other cases can be well described numerically by means of the standard methods with a Hermitian Hamilton operator. This, however, does not mean that the Hamiltonian of an open quantum system is really Hermitian. Quite the contrary; the singular EPs cause, in either case, nonlinear processes in a finite parameter range around their position [9]. These nonlinear processes being inherent in the non-Hermitian formalism, determine the (parametric) evolution of an open quantum system.

The results obtained from calculations for a system that is embedded in only one environment, seem to contradict these statements. That is, such a system can be described by taking into consideration the influences of EPs and EM or by neglecting them and the results are the same. The reason for this unexpected result is that nonlinear processes are able to restore the correct results (corresponding to the calculations with inclusion of EPs and EM) when EPs and EM are explicitly neglected in the calculation. These results feign a good description of the system by means of a Hermitian Hamilton operator [9]. It should be underlined however that this occurs *only* in the case when the system is embedded in no more than one well-defined environment.

New interesting effects occur in systems that are embedded in more than one environment (which is commonly the case). A realistic example is transmission through a small system (e.g., a quantum dot) which needs at least two environments. One of the two environments is related to the incoming flux and the other one to the outgoing flux. In this case, one of the most important new effects is the appearance of coherence [12] in addition to the always existing dissipation in open quantum systems. Coherence is correlated with an enhanced transmission, and is therefore of relevance for applications.

## 3. Information Entropy and Equilibrium States

The properties of open quantum systems depend on many different conditions, mainly simulated by different parameter values. In many studies, the coupling strength ω between the system and environment is kept constant at a sufficiently high level. In this approach, the influence of a possible variation of the value of the coupling strength ω on the results is largely suppressed.

According to the results of many calculations, the main difference between the Hermitian and non-Hermitian formalism of quantum mechanics lies in two effects: the (mathematical) existence of singular EPs [8] and the (physical) possibility of EM of the eigenfunctions [9]. The most challenging question is the following. Do equilibrium states exist in a system with EPs and EM? In other words, do equilibrium states exist in an open quantum system and how they do look like?

In order to find an answer to this question, we will first define the information entropy. For this purpose, let us consider a system consisting of *N* eigenstates of a non-Hermitian Hamiltonian H, the eigenvalues of which are $E_{i}\equiv E_{i}+i/2\;\Gamma_{i}$ [10]. Here, $E_{i}$ stand for the energy of the *i*th eigenstates and $\Gamma_{i}$ for its width, being inversely proportional to the lifetime, $\tau_{i}$, of the state. Let us choose $E_{i}\approx E_{j\neq i}$ and $\Gamma_{i}\neq \Gamma_{j\neq i}$. Following Shannon [13], the information entropy $H_{\mathrm{ent}}$ for this case can be defined by:

(1) $$\begin{array}{c} H_{\mathrm{ent}}=-\sum_{i=1}^{N}\;p_{i}\;\log_{2}\;p_{i}, \end{array}$$

where $p_{i}$ is the probability to find state *i* and $-\log_{2}\;p_{i}$ is the expectation value of the information content of state *i* (its entropy). The value $H_{\mathrm{ent}}$ is maximum when the different $p_{i}$ are equal. In this case, the system is in an equilibrium state.

## 4. Equilibrium State of an Open Quantum System

An open quantum system (normally embedded in more than one environment) can be in an equilibrium state only under two conditions: (i) the system is far from an EP and (ii) EM is taken into account. The first condition arises from the fact that the properties of the system are not stable when the system is near an EP. The second condition corresponds to the fact that EM is inherent in the eigenfunctions $\Phi_{i}$ of the non-Hermitian Hamiltonian H.

It should be repeated here that it is indeed a challenging question whether or not an equilibrium state exists in an open quantum system. This is because it combines two conflicting concepts: the system is open (meaning that its properties are influenced by the environment) and is, nevertheless, expected to be in equilibrium. It is, therefore, no wonder that this question has not been considered, up to now. To overcome this problem, we introduce the concept of maximum entropy. It is meaningful for an open quantum system as will be shown in the following.

Numerical calculations have shown the following (unexpected) result. By tracing the results for a system embedded in two environments, by varying a certain parameter, the eigenfunctions, $\Phi_{i}$, of the non-Hermitian operator, H, can become orthogonal [12]. Thus, the system does not evolve further.

This result can be understood by considering that the evolution of a system described by the non-Hermitian operator H, is driven by nonlinear terms. These nonlinear terms originate from the EPs and are involved in the corresponding Schrödinger equation. They are responsible for the fact that the system does not evolve without limit, instead the evolution of the system occurs up to a certain final state, at which the eigenfunctions of H are orthogonal (instead of biorthogonal).

The calculations further show that the eigenfunctions $\Phi_{i}$ of the final state are strongly mixed in the set of wavefunctions $\Phi_{k}^{0}$ of the individual original states:

(2) $$\begin{array}{c} \Phi_{i}=\frac{1}{N}\;\sum_{k=1}^{N}\;\Phi_{k}^{0}\;\langle \Phi_{k}^{0}|\Phi_{i}\rangle . \end{array}$$

Here $\Phi_{k}^{0}$ are the eigenfunctions of the non-Hermitian Hamiltonian $H_{0}$, which differs from the full operator H by the disappearance of the non-diagonal matrix elements. This means that the $\Phi_{k}^{0}$ are the wavefunctions of the original (unmixed) states and a mixing of the states via the environment is involved in the final $\Phi_{i}$.

According to numerical calculations, when the entropy is maximum, each of the $\Phi_{k}^{0}$ appears in Equation (2) with the same probability, in all the eigenfunctions $\Phi_{i}$ [12]. This means that the final state is an equilibrium state. It should be underlined once more that a general definition of an equilibrium state in an open quantum system is difficult (or even impossible). It is however always possible to find a state with maximum entropy.

Summarizing the results obtained for a system that is embedded in at least two environments, we state the following: (i) the final state of the evolution of the open quantum system (described by a non-Hermitian Hamiltonian H) is an equilibrium state, and (ii) the eigenfunctions of H at the final state of evolution are orthogonal (not biorthogonal).

## 5. Numerical Studies for Concrete Systems versus Evolution of Open Quantum Systems

Let us come back to the question raised in the Introduction; whether (and when) it is necessary to describe a realistic open quantum system by a non-Hermitian Hamilton operator? The results obtained in different studies are sketched above. They show clearly that the answer to this question depends on the conditions under which the system is considered.

From the point of view of numerical results, which will describe special properties of the system, the answer is the following. The system can be well described by a Hermitian Hamiltonian, *H*, when its individual eigenstates are distant from another, so that the different states of the system (almost) do not influence each other. This happens at low level density. The system, however, has to be described by a non-Hermitian Hamiltonian, H, when the influence of singular points (EPs) cannot be neglected in the considered parameter range. This occurs especially at high level density where the different eigenstates are no longer independent of one another.

This means, that the properties of many realistic systems can be described well by means of a Hermitian Hamiltonian, *H*. This statement corresponds to the longtime experience of standard quantum mechanics.

The situation is completely different when general questions, such as the (parametric) evolution of an open quantum system or the formation of an equilibrium state, are considered. In this case, the nonlinear processes caused by the EPs and involved in the non-Hermitian formalism are decisive. Among other outcomes, they are responsible for the fact that an open quantum system (embedded in at least two environments) will finally stop evolving; the evolution occurs up to the formation of a certain final state. The wavefunctions of this final state are orthogonal (not biorthogonal) even though the Hamiltonian of the system is non-Hermitian. The final state is an equilibrium state of the whole system (including its second-order components related to EM). It is different from the equilibrium state of the corresponding closed system.

## 6. Conclusions

Non-Hermitian quantum physics is a fascinating field of research that not only finds an answer to some long-standing problems of quantum physics (for examples, see the Appendix and also Reference [9]). Above all, it justifies the use of standard Hermitian quantum physics for the description of experimental results in many cases. Non-Hermitian quantum physics is therefore nothing but a realistic extension of standard quantum physics.

From a mathematical point of view, non-Hermitian quantum physics is much more complicated than Hermitian quantum physics. An important difference is the existence of nonlinearities arising from the EPs. However, they only play a role in the vicinity of the EPs.

In spite of these differences, the non-Hermitian and Hermitian formalisms show analogous physical features. An example is the existence of equilibrium states. As shown in the present paper, equilibrium states exist not only in closed quantum systems (described by a Hermitian Hamilton operator) but also in open quantum systems (described by a non-Hermitian Hamilton operator), although with certain restrictions. The equilibrium states of an open and a closed system differ, of course, from one another.

## Appendix A. Puzzling Experimental Results

Over the years, different calculations with non-Hermitian Hamiltonians have been performed in order to explain puzzling experimental results. Here, a few of them will be listed.

1. Some years ago, the evolution of the transmission phase, monitored across a sequence of resonance states, was studied experimentally in work where a multi-level quantum dot was embedded into one of the arms of an Aharonov–Bohm interferometer [14,15,16]. These experiments revealed the presence of unexpected regularity in the measured scattering phases (so-called “phase lapses”), when the number of states occupied by electrons in the dot was sufficiently large. While this behavior could not be fully explained within approaches based upon Hermitian quantum theory, it has been established that the phase lapses can be attributed to the non-Hermitian character of this mesoscopic system, and to changes of the system that occur as the number of electrons in the dot is varied [17]. The observed regularity arises from the overlap of the many long-lived states with the short-lived one, all of which are formed in the regime of overlapping resonance states.
2. An example of an environmentally induced transition that is different in character to that described above is the spin swapping observed in a two-spin system embedded in an environment of neighboring spins [18,19,20]. In describing the damped dynamics of the two-spin system, its interaction with its environment may be taken to be inversely proportional to some characteristic time ($\tau_{SE}$), which degrades the spin-swapping oscillations on some “decoherence time” ($\tau_{\phi }$). In the experiment, two distinct dynamical regimes were observed. In the first of these, the expected proportionalities ω∝b and $\tau_{\phi }\propto \tau_{SE}$ were found as the interaction strength, *b*, between the spins was increased. This behavior agrees with Fermi’s golden rule. On exceeding a critical environmental interaction, however, the swapping was instead found to freeze while the decoherence rate dropped according to $\tau_{\phi }^{-1}\propto b^{2}\;\tau_{SE}$. The transition between these two dynamical regimes was not smooth, but rather had the characteristics of critical phenomenon, occurring once ω becomes imaginary. For such conditions, damping of the spin motion decreases with increased coupling to the environment, in marked contrast to the behavior obtained when ω is real. The observed results are related, in References [18,19,20], to the non-Hermitian Hamiltonian describing the system and to the presence of an EP.
3. The high efficiency of the photosynthesis process (used by plants to convert light energy in reaction centers into chemical energy) is not understood in Hermitian quantum physics [21,22,23,24,25]. Using the formalism for the description of open quantum systems by means of a non-Hermitian Hamilton operator, fluctuations of the cross section near singular points (EPs) are shown to play the decisive role [26]. The fluctuations appear in a natural manner, without any excitation of the internal degrees of freedom of the system. They therefore occur with high efficiency and very quickly. The excitation of resonance states of the system by means of these fluctuations (being the second step of the whole process) takes place much slower than the first one, because it involves the excitation of the internal degrees of freedom of the system. This two-step process as a whole is highly efficient and the decay is bi-exponential. The characteristic features of the process obtained from analytical and numerical studies, are the same as those of light harvesting in photosynthetic organisms [26].
4. Atomic systems can be used to store light and to thus act as a quantum memory. According to experimental results, optical storage can be achieved via stopped light [27]. Recently, this interesting phenomenon has been related to non-Hermitian quantum physics. It has been revealed that light stops at exceptional points (EPs) [28]. The authors of Reference [28] restrict their study to parity-time (PT)-symmetricoptical waveguides. This restriction is, however, not necessary. The phenomenon is rather characteristic of non-Hermitian quantum physics; the external mixing (EM) of the eigenfunctions of a non-Hermitian Hamilton operator becomes infinitely large at an exceptional point (EP) [9], such that any interaction with an external source (such as light) vanishes in approaching an EP.
